# The ROS Scavenger, NAC, Regulates Hepatic Vα14*i*NKT Cells Signaling during Fas mAb-Dependent Fulminant Liver Failure

**DOI:** 10.1371/journal.pone.0038051

**Published:** 2012-06-06

**Authors:** Isaac Downs, Jianfeng Liu, Tak Yee Aw, Patrick A. Adegboyega, Maureen N. Ajuebor

**Affiliations:** 1 Department of Molecular and Cellular Physiology, Louisiana State University Health Sciences Center-Shreveport, Shreveport, Louisiana, United States of America; 2 Department of Pathology, Louisiana State University Health Sciences Center-Shreveport, Shreveport, Louisiana, United States of America; University of Hong Kong, Hong Kong

## Abstract

Uncontrolled systemic activation of the immune system is an early initiating event that leads to development of acute fulminant liver failure (FLF) in mice after treatment with agonistic Fas mAb. In this study, we demonstrate that treatment of mice with N-acetylcysteine (NAC), an ROS scavenger and glutathione (GSH) precursor, almost completely abolished Fas mAb-induced FLF through suppression of Vα14*i*NKT cell activation, IFN-γ signaling, apoptosis and nitrotyrosine formation in liver. In addition, enrichment of the liver with GSH due to Vα14*i*NKT cells deficiency, induced an anti-inflammatory response in the liver of Jα18^−/−^ mice that inhibited apoptosis, nitrotyrosine formation, IFN-γ signaling and effector functions. In summary, we propose a novel and previously unrecognized pro-inflammatory and pro-apoptotic role for endogenous ROS in stimulating Th1 signaling in Vα14*i*NKT cells to promote the development of FLF. Therefore, our study provides critical new insights into how NAC, a ROS scavenger, regulates Th1 signaling in intrahepatic Vα14*i*NKT cells to impact inflammatory and pathological responses.

## Introduction

Fas (CD95), a 45-kDa type I membrane protein, is expressed on numerous cell types including lymphoid cells (NK cells, T cells, Vα14*i*NKT cells) [1], [2], [3], [4], [5] and non-lymphoid cells such as hepatocytes [1], [2], [3]. Fas, a member of the TNF receptor superfamily, plays a vital role in regulating apoptosis in many cell types and is typically stimulated by FasL or agonistic Fas mAb [1], [2]. Upon ligand binding, Fas-associated protein with death domain and procaspase 8 are recruited to initiate caspase 8 proteolytic autocleavage, leading to activation of the effector caspase, caspase 3, and ultimately cell death [1], [2]. Fas activation is often observed in diseases affecting many organ systems including heart, lung and kidney. Of significant relevance, Fas activation is a primary trigger for apoptotic death of hepatocytes [1], [2]. The fundamental concept that the liver is highly sensitive to Fas-mediated apoptosis was first demonstrated in 1993 by Ogasawara and colleagues [1] where systemic administration of agonistic Fas mAb (Jo2) caused acute FLF, and ultimately mice mortality within a few hours due to diffuse hemorrhage and massive apoptosis of hepatocytes [1]. Although Fas activation is widely associated with caspase-mediated cell death, growing evidence have increasingly highlighted an important pro-inflammatory role for Fas in promoting NF-κB/AP-1 activation [6], [7], [8], chemokine/cytokine production [6], [7], [9] and leukocyte infiltration [6], [7], [9] in tissue sites.

Vα14*i*NKT cells are thymic-derived innate T lymphocytes that express a highly restricted TCR characterized by a Vα14-Jα18 rearrangement [10]. Distinct from conventional T cells, Vα14*i*NKT cells respond to glycolipid antigens presented by CD1d bearing antigen presenting cells [11]. Since the identity of the endogenous glycolipid ligand that is responsible for Vα14*i*NKT cell selection and development in the thymus remains elusive [12], [13], [14], characterization of several exogenous glycolipids that Vα14*i*NKT cells respond to, α-galactosylceramide (GalCer) and its derivatives, has greatly facilitated the research into the functional role of Vα14*i*NKT cells in health and diseases [13], [14]. Notably, CD1d tetramers loaded with the prototypical synthetic glycolipid antigen, α-GalCer, has been used to reveal that murine liver has the highest frequency of resident Vα14*i*NKT cells [15], [16]. Vα14*i*NKT cells are activated in a TCR-dependent manner by lipids presented by CD1d [17] or by TCR independent mechanisms involving toll like receptors [17], [18], [19], [20]. Following activation, Vα14*i*NKT cells may display cytotoxicity via Fas-FasL and TRAIL-dependent death pathways [17], much like NK cells [21]. However, their major function is thought to be rapid release of copious amounts of immunopolarizing cytokines (including IFN-γ, IL-4 and TNF-α) and chemokines leading to stimulation or suppression of immune responses [17]. Through these mediators, activated Vα14*i*NKT cells can “bridge” the innate and adaptive immune systems by interacting with and transactivating immune cells [22], [23], [24]. This ability to respond rapidly at the onset of the immune response underscores the role of Vα14*i*NKT cells in immune response regulation. Consequently, Vα14*i*NKT cells have been demonstrated to play a critical role in several immune processes, from prevention of inflammation and autoimmunity to protection against various pathogens, including bacteria and viruses [25], [26].

We recently demonstrated that Jα18^−/−^ mice, which are specifically deficient in Vα14*i*NKT cells, are highly resistant to agonistic Fas mAb-induced acute FLF [5]. But the endogenous mechanism(s) regulating the pathophysiological activities of hepatic Vα14*i*NKT cells are not known. In the present study, we hypothesized that activation of the Fas receptor on liver parenchymal cells, hepatocytes, by agonistic Fas mAb, initiates an inflammatory response that induces an endogenous mediator, possibly ROS, to regulate the pathophysiological effects of intrahepatic Vα14*i*NKT cell signaling during acute FLF.

## Materials and Methods

### Mice

Male C57BL/6 mice and IFN-γ^−/−^ mice (on C57BL/6 background) were purchased from the Jackson Laboratory (Bar Harbor, ME). Breeding pairs of Jα18^−/−^ mice (on C57BL/6 background) were kindly provided by Dr. M. Taniguchi (RIKEN Research Center for Allergy & Immunology, Yokohoma, Japan) [27] and bred in a pathogen-free breeding facility at LSUHSC-Shreveport [5], [20]. All mice were fed a standard chow pellet diet, had free access to water and were maintained on a 12 h light/dark cycle in a pathogen-free facility. All experiments were conducted in accordance with National Institutes of Health and LSUHSC-Shreveport guidelines for animal care. All experiments were approved by LSUHSC-Shreveport Animal Care and User Committee (Proposal #: P11-043).

### Agonistic Fas (CD95) mAb-mediated FLF

Agonistic Fas mAb (clone Jo2; 0.5 µg/g of body weight; BD Pharmingen; San Diego, CA) was administered intraperitoneally to mice for 4.5 h to induce liver injury as we recently described [5]. This dose of Fas mAb does not cause mice mortality. Control mice received an equivalent volume of sterile PBS [28], [29], [30]. At indicated time-point, mice were anesthetized with a mixture of xylazine and ketamine hydrochloride and blood serum collected. All livers were then perfused with ice-cold sterile PBS (to remove blood elements) and harvested for experimental assays described below. In some experiments, mice were treated with a single dose of freshly prepared ROS scavenger, N-acetylcysteine (NAC; 300 mg/kg, i.p.; Sigma) [20], [31] immediately after Fas mAb treatment.

### Glutathione (GSH) Measurement

Perfused livers were snap-frozen in liquid nitrogen immediately after excision from mice. Total GSH in liver was determined in trichloroacetic acid supernatants by high-performance liquid chromatography (HPLC) using a modified protocol of Reed *et al*. [32] as we previously described [33], [34]. Briefly, experimental samples were derivatized with 6 mM iodoacetic acid and 1% 2,4-dinitrofluorobenzene to yield the *S*-carboxymethyl and 2,4-dinitrophenyl derivatives, respectively. Separation of GSH derivative was performed on a 250×4.6-mm Alltech Lichrosorb NH_2_ 10-µm column using a Shimadzu HPLC system. Proteins in the acid pellet were solubilized in 0.1 M NaOH, and protein was determined using the Bio-Rad Protein Assay kit (Bio-Rad, Hercules, CA). GSH concentration was determined by comparison with purified GSH standards derivatized in the same manner.

### Western Blot Analysis

Perfused mice livers were homogenized in RIPA buffer (50 mM Tris-HCl pH 7.4, 1% Nonidet-P40, 0.25% Sodium deoxycholate, 150 mM NaCl, 1 mM EDTA, 1 mM Dithiothreitol and protease inhibitors). Equal volumes of 2x sample buffer were added to liver protein extract. Next, liver protein samples (50 µg/lane) were fractionated by SDS-PAGE and then transfered onto PVDF membrane (Thermo Scientific; Rockford, IL). After which, membranes were blocked with 5% fat-free milk for 1 h at room temperature followed by overnight incubation with the following primary antibodies at depicted dilutions/concentrations: active caspase 3 (1∶1000); pSTAT-1 (1∶500); T-bet (1∶1000), GAPDH (1∶1000) and Nitrotyrosine (1∶1000). Membranes were then counterstained with corresponding horseradish peroxidase-conjugated secondary antibodies for visualization by Pierce ECL western blotting reagent (Thermo Scientific). Each membrane was stripped in buffer (0.5 mM Tris-HCl pH 6.8, 10% SDS, 0.08% Mercaptoethanol) and probed for GAPDH to verify equal protein loading in samples. Active caspase 3 Ab (clone 269518) was obtained from R & D systems (Minneapolis, MN) whereas pSTAT-1 (Tyr701) Ab was supplied by BD Pharmingen. Antibodies for T-bet (4B10) and GAPDH were all purchased from Santa-Cruz Biotech (Santa-Cruz, CA) and Nitrotyrosine mAb (clone HM11) was supplied by Invitrogen (Camarillo, CA).

### Biochemical and Histological Liver Injury

Acute liver injury was evaluated by biochemical and histological means. Biochemical assessment of liver damage was determined by serum levels of the liver enzyme, alanine aminotransferase (ALT) using a commercial kit (Thermo Electron, Waltham) [5], [35]. For histological evaluation, paraffin embedded liver sections (5 µm thick) were deparaffinized, stained with H & E according to standard protocols and then analyzed by light microscopy in a blinded fashion by a pathologist (PAA). The degree of inflammation in the liver and hepatocyte damage was graded as none (0), mild (<25%), moderate (25%–50%) and severe (>50%) using a combination of indices: severity of the inflammation and degree of hepatocyte degenerative changes including hepatocyte necrosis, hemorrhage and frequency of acidophilic bodies [5], [35].

### In Situ Analysis of Liver Apoptosis Using TUNEL

Paraffin-embedded liver sections were dewaxed in xylene and rehydrated by passage through a graded series of ethanol solutions, and then PBS. Sections were permeabilized with proteinase K (20 µg/ml in 10 mM Tris-HCl, pH 7.4–8.0) at 37°C for 15 min, washed and then stained with fluorescein nucleotide mixture with terminal deoxynucleotidyl transferase (TdT) from In Situ Cell Death Detection kit (Roche Applied Science; Indianapolis, IN). Sections were viewed and photographed using standard fluorescent microscopic techniques.

**Figure 1:**
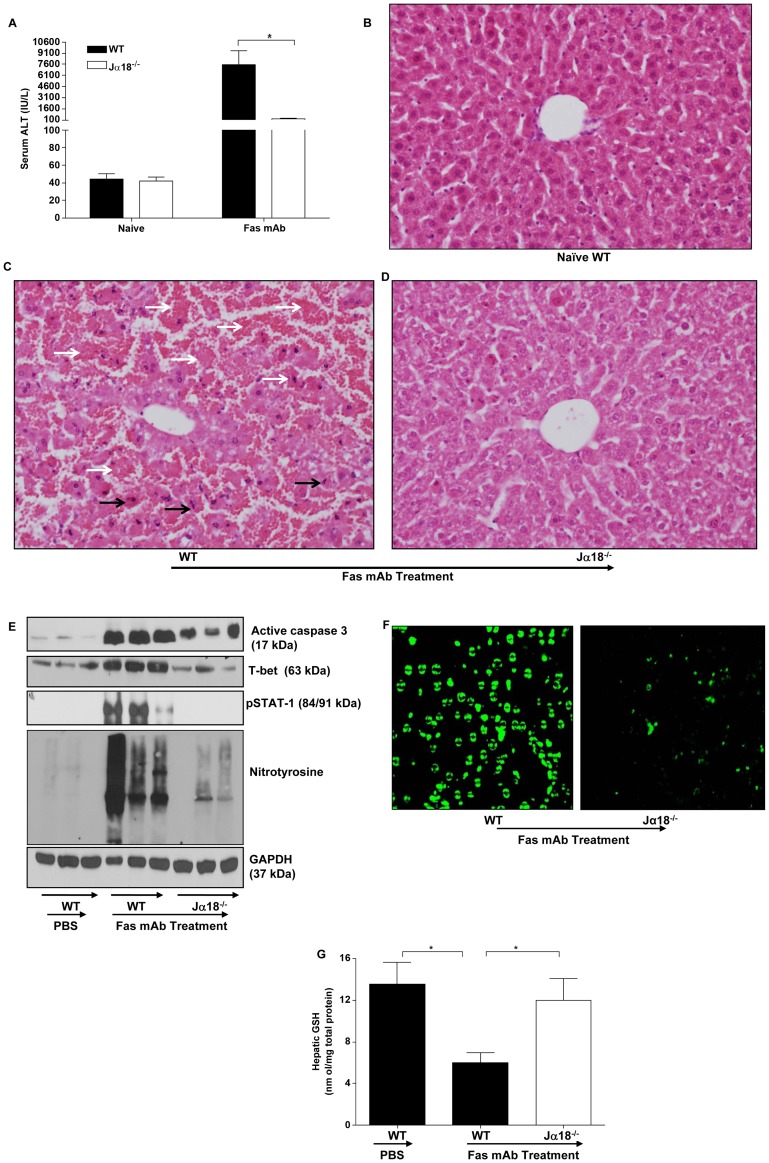
Th1 differentiating signaling in the liver is dysregulated by Vα14*i*NKT cells deficiency during Fas mAb-induced FLF. (**a**) Serum ALT levels of naïve WT mice, Fas mAb-treated WT and Jα18^−/−^ mice at 4.5 h. (**b–d**) H & E staining of liver sections from naïve WT mice, Fas mAb-treated WT and Jα18^−/−^ mice at 4.5 h. Livers from Fas mAb-treated WT mice (**c**) showed extensive damage with destruction of hepatocytes and distortion of normal liver architecture. The hepatocytes show hemorrhagic necrosis (white arrows) and characteristic signs of apoptosis (black arrows) including chromatin condensation and cellular shrinkage. In comparison, livers from Fas mAb-treated Jα18^−/−^ mice showed only minimal damage and retained the normal architecture (**d**). Liver from a naïve WT mouse is illustrated in (**b**) for comparison. (**e**) Western blot analysis of active caspase 3, T-bet, pSTAT-1, nitrotyrosine and GAPDH expression in the liver of PBS-treated WT mice and agonistic Fas mAb-treated WT and Jα18^−/−^ mice at 4.5 h. (**f**) TUNEL staining of liver sections from WT and Jα18^−/−^ mice at 4.5 h after Fas mAb injection in which WT mice showed intense TUNEL staining characteristic of apoptosis whereas Jα18^−/−^ mice showed less/reduced TUNEL staining. (**g**) HPLC measurement of hepatic GSH levels in PBS-treated WT mice and Fas mAb-treated WT and Jα18^−/−^ mice at 4.5 h. Figure S1 in a and g are presented as mean ± s.e.m with *n* = 5 mice/group; *P<0.05 by one-way analysis of variance followed by Newman-Kuels post hoc test. All experiments were conducted twice.

**Figure 2:**
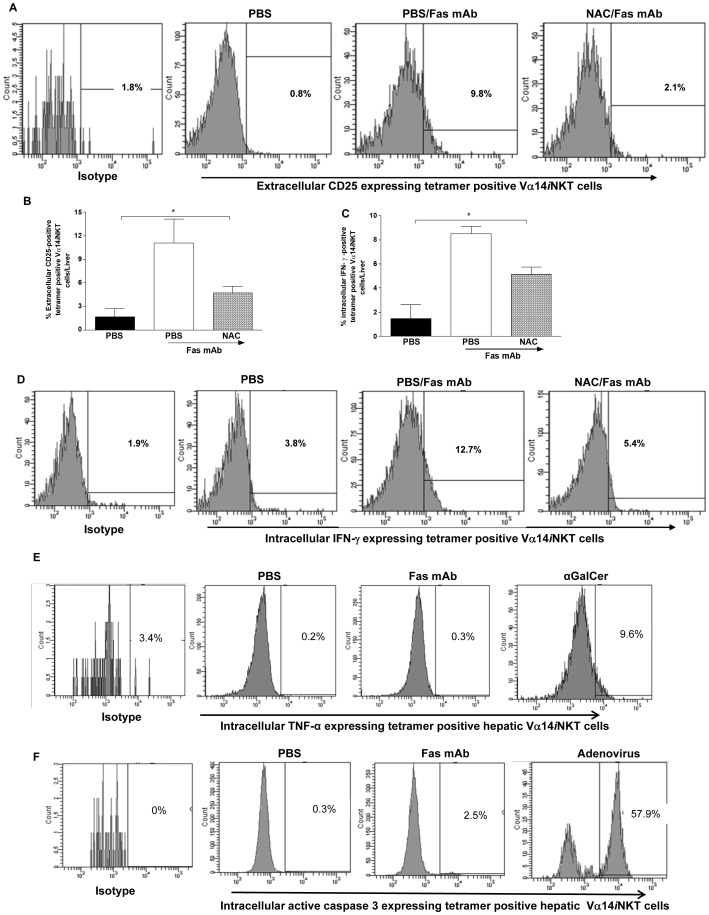
Effect of NAC treatment on intrahepatic Vα14*i*NKT cell activation during agonistic Fas mAb-induced FLF. Representative FACS histograms of extracellular CD25 (**a**), intracellular IFN-γ (**d**), intracellular TNF-α (**e;** αGalcer used as a positive control) and intracellular active caspase 3 (**f**; Adenovirus used as a positive control) expression levels by intrahepatic Vα14*i*NKT cells at 4.5 h after PBS or agonistic Fas mAb treatment. All experiments were performed twice. Figure S1 in **b** and **c** are presented as mean ± s.e.m with *n* = 4 mice/group; *P<0.05 by one-way analysis of variance followed by Newman-Kuels post hoc test.

### Hepatic Lymphocytes Isolation and Flow Cytometry

Hepatic lymphocytes were isolated using our published protocols [16], [20], [35]. To specifically identify Vα14*i*NKT cells by flow cytometry, isolated hepatic lymphocytes were preincubated with anti-mouse CD16/32 mAb (clone 2.4G2; BD Pharmingen) to block FcγRs and then incubated simultaneously with fluorochrome-labeled TCRβ mAb (clone H57-597; eBiosciences, San Diego, CA) and fluorochrome-labeled Vα14*i*NKT cell tetramer (CD1d-PBS57; NIH Tetramer Core Facility, Atlanta) [5], [20], [35]. CD25 expression on the surface of tetramer positive hepatic Vα14*i*NKT cells was determined by FACS after staining with fluorochrome-labeled murine CD25 mAb (clone PC61.5; eBiosciences). For measurement of intracellular IFN-γ, TNF-α and active caspase 3, tetramer positive Vα14*i*NKT cells were first permeabilized using the Cytoperm/fix kit (BD Pharmingen) and then stained with either fluorochrome-labeled murine IFN-γ mAb (clone XMG1.2; BD Pharmingen) [5], [35], fluorochrome-labeled murine TNF-α mAb (clone MP6-XT22; eBioscience) [20] or fluorochrome-labeled active caspase 3 mAb (clone C92-605; BD Pharmingen) [5], [16], [20]. In all experiments, cells were analyzed directly *ex vivo* without cell culture treatment with brefeldin A or monensin. Corresponding isotype antibody/tetramer was used to set analysis gates. In addition, viable lymphocyte populations were gated using forward and side scatter characteristics and data analyzed using the FACS Calibur and FACS Scan Diva software (BD Pharmingen).

### Statistical Analysis

All data are shown as mean ± SEM. For comparisons of means between 2 experimental groups, a Student unpaired *t* test was used. Comparison among three or more experimental groups was performed using a one-way ANOVA, followed by Newman-Kuels post hoc test. A value of *p*<0.05 was considered significant.

**Figure 3:**
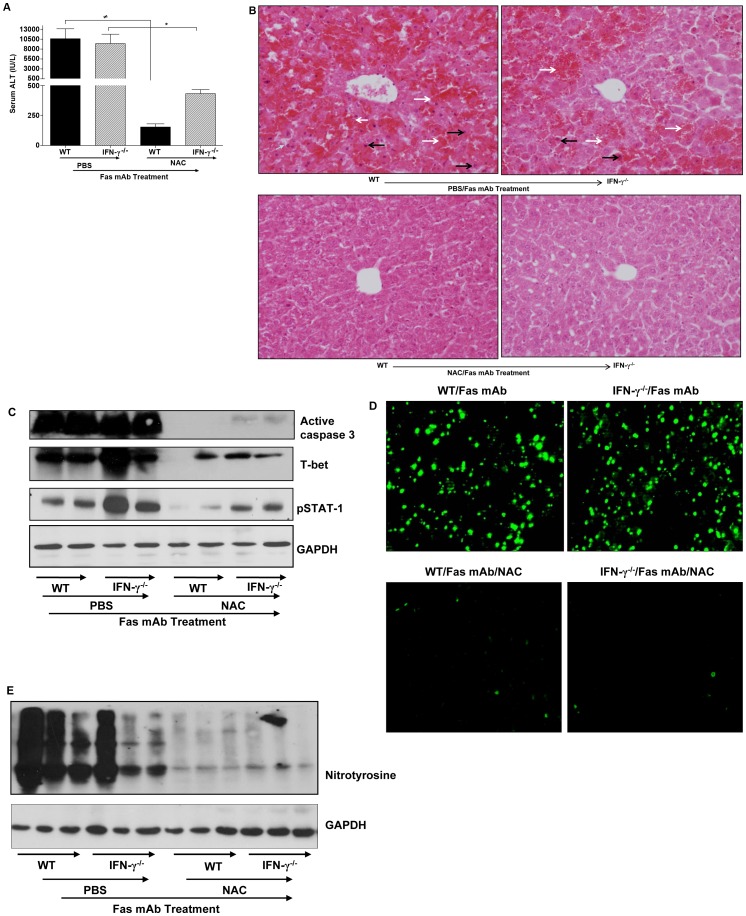
NAC therapy ameliorates agonistic Fas mAb-induced FLF during IFN-γ deficiency. (**a**) Serum ALT levels in WT and IFN-γ^−/−^ mice after PBS or NAC treatment during agonistic Fas mAb-induced FLF. (**b**) H & E staining of liver sections of WT and IFN-γ^−/−^ mice after PBS or NAC treatment during agonistic Fas mAb-induced FLF. As shown in **top panel**, livers from Fas mAb-treated WT and IFN-γ^−/−^ mice displayed widespread hepatocyte damage including hemorrhagic necrosis (white arrows) and apoptosis (black arrows) that distorted normal liver architecture. In contrast, liver sections of WT and IFN-γ^−/−^ mice treated with NAC during Fas mAb-induced FLF (**bottom panel**) showed reduced hepatocyte damage and retained near normal architecture. (**c & e**) Western blot analysis of hepatic active caspase 3, T-bet, pSTAT-1 expression levels and nitrotyrosine formation in WT and IFN-γ^−/−^ mice after PBS or NAC treatment during Fas mAb-induced FLF. (**d**) TUNEL staining of liver sections from WT and IFN-γ^−/−^ mice treated with PBS during Fas mAb-induced FLF showed intense TUNEL staining characteristic of apoptosis whereas WT and IFN-γ^−/−^ mice treated with NAC mice showed minimal TUNEL staining. Figure S1 in **a** are presented as mean ± s.e.m with *n* = 3–6 mice/group. *P<0.05, ^≠^ *P*<0.05 by one-way analysis of variance followed by Newman-Kuels post hoc test. All experiments were performed twice.

## Results

### Resistance of Vα14iNKT Cells Deficient Mice to FLF is Associated with Decreased Th1 Differentiating Signaling in Liver

We first confirmed our recent observation [5] that the presence of hepatic Vα14*i*NKT cells promote acute FLF in response to agonistic Fas mAb treatment. Specifically, we found that Fas mAb administration into WT mice caused a significant increase in serum ALT level whereas Jα18^−/−^ mice were highly resistant to acute FLF as reflected by almost complete suppression (>90% reduction) of serum ALT (Figure 1A). In parallel, liver sections from WT mice exhibited extensive hepatocyte apoptosis and necrotic damage following Fas mAb treatment relative to livers from Jα18^−/−^ mice which displayed mild hepatocyte damage (Figure 1C and D). Specifically, the degree of hepatic inflammation and hepatocyte damage in WT mice after Fas mAb treatment was graded as severe (>50%) relative to mild (<25%) in Jα18^−/−^ mice. As expected, normal serum ALT levels was observed in both naive WT and J Jα18^−/−^ mice (Figure 1A). In the present study, we provide new data demonstrating that resistance of Jα18^−/−^ mice to FLF was associated with a dramatic decrease in hepatic apoptosis as revealed by reduced expression of active caspase 3 and TUNEL staining in the liver (Figure 1E and 1F). The finding that active caspase 3 expression was not completely suppressed in Jα18^−/−^ mice after Fas mAb treatment suggests that other hepatic cells apart from intrahepatic Vα14*i*NKT cells may also contribute to apoptosis. It is notable that reduced susceptibility of Jα18^−/−^ mice to FLF was also accompanied by striking reductions in hepatic expression of Th1 differentiating signaling molecules, pSTAT-1 and T-bet (Figure 1E). To determine whether oxidative and nitrosative stress may also contribute to the development of FLF, we measured nitrotyrosine formation (a product of nitrosative stress) and the ROS scavenger, GSH. We observed a striking increase in nitrotyrosine formation in the liver of WT mice but not Jα18^−/−^ mice after Fas mAb administration (Figure 1E). Remarkably, we also found that Fas mAb-mediated FLF in WT mice caused a significant decrease in hepatic GSH (relative to PBS-treated WT mice), but GSH levels were restored in the absence of Vα14*i*NKT cells (i.e. in Jα18^−/−^ mice) during mild FLF to levels seen in PBS-treated WT mice (Figure 1G).

**Figure 4:**
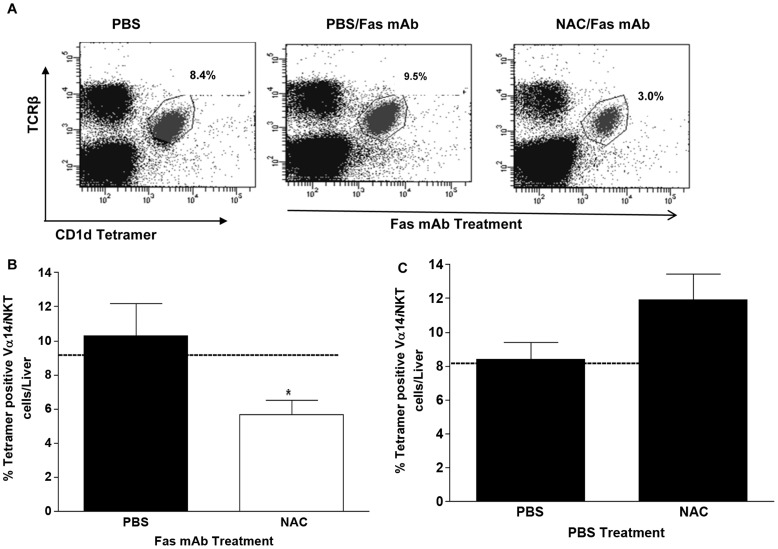
Effect of NAC treatment on intrahepatic Vα14*i*NKT cell accumulation during Fas mAb-induced FLF. (**a**) Representative FACS dot plot of Vα14*i*NKT cells levels in the liver after PBS or NAC treatment during Fas mAb-induced FLF. (**b**) FACS analysis of Vα14*i*NKT cells level in the liver after PBS or NAC treatment in response to Fas mAb-induced FLF. (**c**) FACS analysis of Vα14*i*NKT cells level in the liver after PBS or NAC treatment only (i.e. in the absence of agonistic Fas mAb). Results in **b** and **c** are shown as mean ± s.e.m with *n* = 4–6 mice/group with *P<0.05 by Student’s unpaired *t* test. All experiments were conducted twice. Broken lines denote levels in untreated mice.

### Agonistic Fas mAb Promotes Intrahepatic Vα14iNKT Cell Activation

We next verified by flow cytometry that hepatic Vα14*i*NKT cells were activated following agonistic Fas mAb administration in WT mice as denoted by upregulation of the activation marker, CD25, on cell surface (Figure 2A and B) and by increased intracellular IFN-γ expression by hepatic Vα14*i*NKT cells (Figure 2C and D). In addition, we established that the ROS scavenger, NAC, effectively suppressed hepatic Vα14*i*NKT cells CD25 and IFN-γ expression in WT mice during Fas mAb-mediated FLF (Figure 2A, B, C, D). Although CD25 expression by hepatic Vα14*i*NKT cells in NAC-treated WT mice during Fas mAb-mediated FLF was 2-fold higher than PBS control, it was not significant (Figure 2B). In contrast, hepatic Vα14*i*NKT cells IFN-γ expression in NAC-treated WT mice during Fas mAb-mediated FLF was significantly higher (i.e. 3-fold) than PBS control (Figure 2C). Moreover, the number of CD25-positive cells but not IFN-γ positive cells in the liver of WT mice after NAC/Fas mAb treatment was significantly higher than PBS control (Figure S1). It is noteworthy that Vα14*i*NKT cells from the liver of Fas mAb-treated WT mice lack intracellular TNF-α (Figure 2E) and active caspase 3 (Figure 2F).

**Figure 5:**
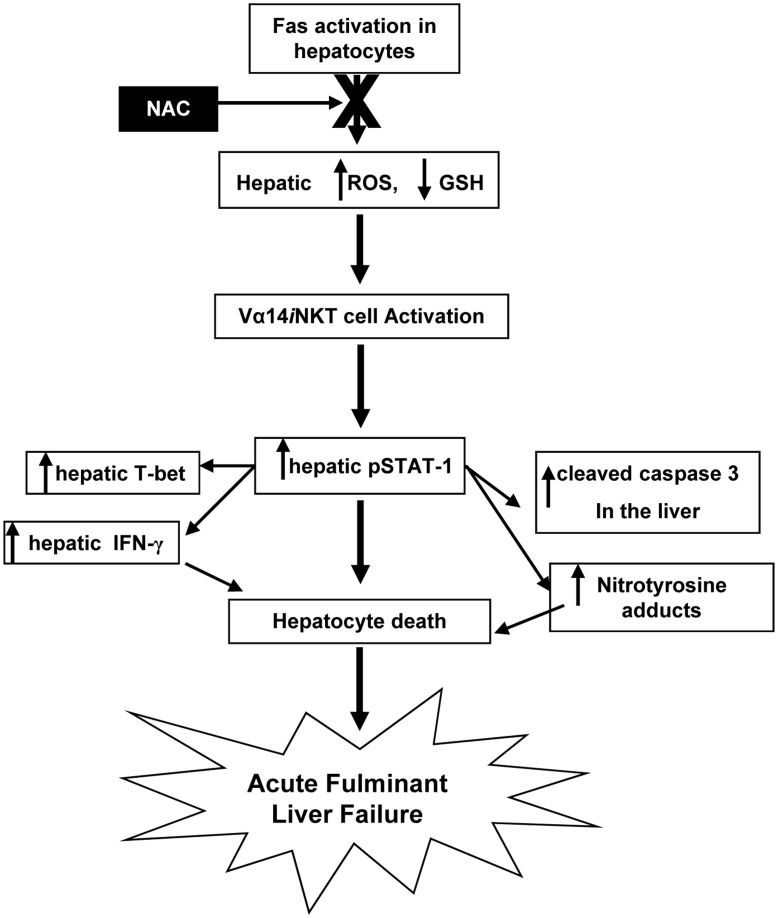
A proposed model depicting the sequential molecular and cellular events of NAC therapy regulation of Vα14*i*NKT cells signaling in the liver during Fas mAb-induced FLF. An endogenous mediator inhibited by NAC (possibly ROS) mediates Fas mAb-dependent FLF by promoting intrahepatic Vα14*i*NKT cells signaling, upregulation of pSTAT-1 and pSTAT-1-regulated genes, caspase 3 and T-bet, induction of hepatocyte damage and fatal/lethal immunopathological events in the liver that ultimately leads to FLF.

### Pathophysiological Role of IFN-γ During Fas mAb-dependent FLF

In view of our preceding findings, we next treated WT and IFN-γ^−/−^ mice with agonistic Fas mAb to evaluate whether IFN-γ is an essential and direct participant in FLF. As shown in Figure 3A, both WT and IFN-γ^−/−^ mice were similarly susceptible to acute FLF since serum ALT levels in both mice strains were comparable. In correlation, histological evaluation of liver sections showed that the degree of hepatic inflammation and hepatocyte damage in both strains of mice was severe (>50%; Figure 3B, top panels). Histological scoring criteria was based on the severity/magnitude of inflammation, and the degree of hepatocyte degenerative changes including hepatocyte necrosis, hemorrhage and frequency of acidophilic bodies [5], [35]. Likewise, active caspase 3 expression and tunnel staining in the liver during IFN-γ deficiency was comparable to levels in WT mice during Fas mAb-mediated acute FLF (Figure 3C and D). It is also notable that the strong expression levels of pSTAT-1 and T-bet in the liver of WT mice during FLF was not suppressed by IFN-γ deficiency (Figure 3C). Likewise, nitrotyrosine formation in the liver of WT mice was comparable to IFN-γ^−/−^ mice during Fas mAb-mediated acute FLF (Figure 3E). These data strongly suggests that IFN-γ may not be a key and/or direct mediator of FLF in response to agonistic Fas mAb treatment.

### NAC Therapy Alleviates Fas mAb-dependent FLF During IFN-γ Deficiency

We demonstrated in Figure 1G that resistance of Jα18^−/−^ mice to hepatic failure may be associated with elevated GSH levels. GSH has been reported to ameliorate Fas mAb-dependent FLF [30], [36]. For this reason, we evaluated the effects of NAC, a GSH precursor and ROS scavenger, on the development of FLF in WT and IFN-γ^−/−^ mice in response to agonistic Fas mAb. Importantly, we observed that WT mice were strikingly resistant to Fas mAb-dependent FLF following NAC treatment as denoted by considerably lower ALT levels relative to Fas mAb-treated WT mice given PBS (Figure 3A). Equally significant, FLF in IFN-γ^−/−^ mice was almost completely resolved by NAC therapy relative to Fas mAb-treated IFN-γ^−/−^ mice administered PBS (Figure 3A). In parallel, histological injury in both strains of mice was found to be minimal (Figure 3B) after NAC therapy since the scale of hepatic inflammation and hepatocyte damage in both strains of mice were graded as very mild to none. Notably, NAC therapy in WT and IFN-γ^−/−^ mice also markedly suppressed hepatic apoptosis as revealed by active caspase 3 (Figure 3C) and TUNEL staining (Figure 3D). Additionally, pSTAT-1 (Figure 3C), T-bet (Figure 3C) expression and nitrotyrosine formation (Figure 3E) in the liver of WT and IFN-γ^−/−^ mice were all suppressed by NAC therapy. These findings suggest a fundamental role for endogenous ROS in regulating Th1 differentiating signaling and nitrosative stress in the liver during Fas mAb-dependent FLF.

### NAC Therapy Prevents Hepatic Vα14iNKT Cell Accumulation During FLF

Given the anti-inflammatory effects of NAC therapy on Fas mAb-dependent FLF and Th1 differentiating signaling, we next determined whether NAC may also inhibit intrahepatic Vα14*i*NKT cell accumulation. Indeed, we found that NAC therapy effectively diminished the frequency of Vα14*i*NKT cells in the liver of WT mice undergoing acute FLF (Figure 4A and B). It is also notable that the frequency of Vα14*i*NKT cells in the liver of WT mice treated only with NAC (i.e. in the absence of agonistic Fas mAb) did not significantly differ from WT mice given only PBS (Figure 4C), suggesting that endogenous ROS produced in inflamed liver during agonistic Fas mAb-mediated FLF may be driving the effects seen on intrahepatic Vα14*i*NKT cell accumulation.

### Effects of NAC Therapy on Vα14iNKT TCR Downregulation

These experiments were designed to determine whether the suppressive effects of NAC therapy on Vα14*i*NKT cells accumulation in the liver during agonistic Fas mAb-mediated FLF could be due to down-modulation of surface TCR. Our results showed that surface TCRβ on Vα14*i*NKT cells was not downregulated by NAC therapy during Fas mAb-induced FLF since the geometric mean fluorescence intensity (MFI) of surface TCRβ after NAC treatment was comparable to WT mice administered PBS (MFI: 1548±354 in WT/NAC/Fas mAb relative to 1539±343 in WT/PBS/Fas mAb; *n* = 6 mice/group).

## Discussion

Engagement of the Fas receptor typically leads to apoptosis [1], [2], [3]. The importance of the Fas/FasL system in hepatic apoptosis has been convincingly demonstrated in both experimental and clinical liver injury models including viral and autoimmune hepatitis, alcoholic liver disease and acute liver failure [1], [2], [3], [5], [37], [38]. Therefore, strategies for downregulating the Fas/FasL system might have therapeutic value in the treatment of these human diseases. In addition to its role in caspase-mediated cell death, emerging studies have increasingly proposed an inflammatory role for agonistic Fas mAb in stimulating intracellular signaling pathways in target cells, such as hepatocytes, astrocytes and epithelial cells, leading to NF-κB and/or AP-1 activation [6], [7], [8], chemokine/cytokine production [6], [7] and leukocyte infiltration [6], [7], [9] in tissue sites. Vα14iNKT cells represent a critical link between the innate and adaptive immune systems and play an important immunoregulatory role in hepatic, cardiovascular, infectious and autoimmune diseases as well as in tumor immunity. We recently demonstrated that mice deficient in Vα14*i*NKT cells (i.e. Jα18^−/−^ mice) are highly resistant to acute FLF in response to Fas mAb treatment [5]. But, there are notable deficiencies in our knowledge regarding whether; (i) agonistic Fas mAb directly stimulates intrahepatic Vα14*i*NKT cells to induce effector functions or (ii) inflammatory mediator(s) are produced in the liver in response to agonistic Fas mAb treatment to alter/regulate the biological/functional effects of intrahepatic Vα14*i*NKT cells. The current study highlights a novel dual pro-inflammatory and pro-apoptotic role for endogenous ROS in mediating agonistic Fas mAb-dependent acute FLF by promoting intrahepatic Vα14*i*NKT cell activation and effector functions.

During inflammatory responses, Vα14*i*NKT cells are rapidly activated by TCR-dependent and independent mechanisms [17], [18], [19], [20] to produce significant amounts of immunopolarizing cytokines including the Th1 cytokine, IFN-γ [16] and TNF-α [20]. For this reason, we initially ascertained the activation status of intrahepatic Vα14*i*NKT cells in response to agonistic Fas mAb treatment. We observed by FACS analysis that hepatic Vα14*i*NKT cells are activated to upregulate extracellular CD25 and intracellular IFN-γ expression but not TNF-α. Our approach of using intracellular IFN-γ production and/or extracellular CD25 expression to denote Vα14*i*NKT cell activation is widely supported by multiple studies from our laboratory [5], [20] and others [22], [39], [40], [41], [42], [43], [44], [45]. Since many of the effects of IFN-γ are STAT-1 and T-bet dependent [46], [47], [48], we also determined by western blotting if these Th1 differentiating signaling molecules are differentially regulated in the presence and absence of Vα14*i*NKT cells following Fas mAb administration. Consistent with this notion, we found that pSTAT-1 and T-bet levels in the liver were markedly diminished in the absence of Vα14*i*NKT cells. Additionally, markers of apoptosis (i.e. active caspase 3 and TUNEL staining) and nitrosative stress (i.e. nitrotyrosine formation) were suppressed by the deficiency in Vα14*i*NKT cells during Fas mAb-dependent FLF. Therefore, we propose that Vα14*i*NKT cells positively regulates the expression of Th1 differentiating signaling mediators, IFN-γ, STAT-1 and T-bet, in the liver as well as liver apoptosis and nitrosative stress during Fas mAb-dependent FLF.

To provide proof-of-principle that the pro-inflammatory/pathological effects of intrahepatic Vα14*i*NKT cells could be directly mediated by IFN-γ, we examined the effects of IFN-γ deficiency on the development of Fas mAb-dependent FLF. Astonishingly, IFN-γ mutant mice were similarly susceptible to Fas mAb-induced FLF as WT mice. In correlation, a previous study demonstrates that IFN-γ can exert liver inflammation/injury independent of Fas [49]. Furthermore, hepatic apoptosis was not alleviated by IFN-γ deficiency since expression of active caspase 3 and TUNEL positive cells in the liver of IFN-γ mutant mice was comparable to WT mice. Although STAT-1 and T-bet are usually critical to IFN-γ signaling, we found that IFN-γ is dispensable for hepatic induction of pSTAT-1 and T-bet following Fas mAb treatment since their expression was not inhibited by IFN-γ deficiency. In view of the fact that pSTAT-1 is an upstream transcription factor known to induce caspase 3-dependent apoptosis [46], [47] and T-bet activation [46], [48], we speculated that endogenous factor(s) upstream of IFN-γ may be early activator(s) of STAT-1 and T-bet. With this in mind, our subsequent experiments were designed to establish if treating mice with NAC to block ROS, an endogenous mediator produced in the liver (i.e. hepatocytes) during Fas mAb-induced FLF [50], [51], [52], may suppress IFN-γ signaling. Alternatively, NAC may directly inhibit pSTAT-1 and T-bet activation independent of IFN-γ.

There is growing evidence that Fas mAb-induced acute FLF is a result of complex pathophysiological events involving injurious factors such as ROS. For example, studies highlighting the deleterious consequences of GSH depletion in Fas mAb-mediated FLF underscore the importance of this anti-oxidant [30], [36]. Likewise, administration of MnTBAP (a nonpeptidyl mimic of superoxide dismutase) [51] prevents Fas mAb-induced FLF. Further, GSH enrichment alleviates Fas mAb-induced acute FLF [30]. For this reason, we next determined the effect of NAC treatment on IFN-γ signaling during Fas mAb-induced FLF by treating IFN-γ^−/−^ mice with NAC. We demonstrate for the first time that NAC therapy diminished hepatic injury in IFN-γ^−/−^ mice during Fas mAb-dependent FLF probably via suppression of hepatic Th1 signaling molecules (i.e. pSTAT-1 and T-bet), apoptosis and nitrotyrosine formation. However, our observation that pSTAT-1 and T-bet expression in both vehicle and NAC treatment groups in IFN-γ^−/−^ mice was higher than in WT mice suggests a possible inhibitory role of IFN-γ^−^ that may not involve caspase 3. This observation warrants further investigation. Taken together, these studies provide important evidence that NAC therapy regulates IFN-γ signaling and effector functions during Fas mAb-induced FLF. An important question our study also addressed is why Jα18^−/−^ mice are resistant to development of FLF and hepatic apoptosis following Fas mAb administration. It is notable that Fas stimulation induces hepatic GSH depletion by triggering a cellular efflux of reduced GSH [53], [54]. Conversely, preventing Fas induced GSH depletion attenuates apoptosis [30]. In agreement, we observed that GSH levels in the liver of WT mice was significantly depleted in response to agonistic Fas mAb treatment, but completely restored by V Vα14*i*NKT cell deficiency to levels seen in PBS-treated WT mice. As mentioned previously, hepatic apoptosis (as denoted by active caspase 3 and TUNEL staining), pSTAT-1 and T-bet levels in the liver were markedly diminished by Vα14*i*NKT cell deficiency during Fas mAb-induced FLF. On the basis of these findings, we surmised that fortification of hepatic GSH due to absence of Vα14*i*NKT cells generates anti-inflammatory responses that suppresses IFN-γ signaling and effector functions in the liver to prevent/limit the development of FLF and hepatic apoptosis in Jα18^−/−^ mice.

We previously discussed that intrahepatic Vα14*i*NKT cells are activated during agonistic Fas mAb-induced FLF to express extracellular CD25 and intracellular IFN-γ. However, it is not known whether agonistic Fas mAb is capable of directly stimulating intrahepatic Vα14*i*NKT cells. Work by us [5], [16] and others [4], [40], [55] in several animal models have previously demonstrated that intrahepatic Vα14*i*NKT cell activation during inflammatory responses is associated with a significant decline in the frequency of hepatic Vα14*i*NKT cells due to death by apoptosis, a process known as activation-induced cell death (AICD). In the present study, we provide evidence supporting the notion that agonistic Fas mAb is unlikely to directly stimulate intrahepatic Vα14*i*NKT cells to undergo AICD. Specifically, Fas mAb treatment in WT mice did not cause a decline in intrahepatic Vα14*i*NKT cells since the frequency of intrahepatic Vα14*i*NKT cells in Fas mAb-treated WT mice was comparable to PBS-treated WT mice. Furthermore, intrahepatic Vα14*i*NKT cells lack intracellular active caspase 3 upon Fas mAb treatment. Hence, we next assessed the effect of NAC therapy on intrahepatic Vα14*i*NKT cell activation during Fas mAb-induced FLF.

Recently, we showed that NAC therapy inhibits intrahepatic Vα14*i*NKT cell activation during poly I:C-induced liver inflammation [20]. To further explore the effect of NAC therapy on intrahepatic Vα14*i*NKT cell activation in this study, Vα14*i*NKT cells extracellular CD25 and intracellular IFN-γ expression were determined. Significantly, we found that NAC therapy effectively curbed intrahepatic Vα14*i*NKT cell activation (i.e. extracellular CD25 and intracellular IFN-γ expression) in WT mice during Fas mAb-induced FLF. Equally important, liver pathology, hepatic apoptosis and IFN-γ signaling in WT mice were all diminished by NAC treatment during FLF. It is also notable that the frequency of Vα14*i*NKT cells in the liver of WT mice undergoing Fas mAb-induced FLF was also significantly decreased by NAC therapy. In contrast, NAC only treatment of WT mice (i.e. in the absence of agonistic Fas mAb) did not alter the frequency of Vα14*i*NKT cells in the liver relative to WT mice treated only with vehicle (i.e. in the absence of agonistic Fas mAb). These results indicate that NAC effectively suppresses the endogenous mediator produced by inflamed liver to drive the effects seen in intrahepatic Vα14*i*NKT cells during agonistic Fas mAb-mediated FLF. It is generally accepted that the decline/disappearance of Vα14*i*NKT cells during inflammatory responses may result from TCR down-regulation and/or apoptosis [4], [5], [16], [39], [55], [56], [57]. In the current study, we observed that surface TCR on Vα14*i*NKT cells was not down-regulated by NAC therapy during Fas mAb-induced FLF. Furthermore, NAC therapy did not promote apoptosis of intrahepatic Vα14*i*NKT cells following agonistic Fas mAb administration. Although beyond the scope of the current study, it is conceivable that NAC therapy may suppress the production of chemoattractant(s) critical to Vα14*i*NKT cells accumulation in the liver during FLF. This is an area that warrants further investigation.

In summary, the current study reveals new insights into how NAC therapy regulates IFN-γ signaling in Vα14*i*NKT cells to impact inflammatory and pathological responses in the liver (Figure 5) and possibly other tissue sites (such as heart, lung and kidney) where Fas activation is often observed.

## Supporting Information

Figure S1 **Effect of NAC treatment on intrahepatic Vα14** ***i*** **NKT cell CD25 expression during agonistic Fas mAb-induced FLF.** The number of CD25-expressing Vα14*i*NKT cells in the liver after PBS or NAC treatment during Fas mAb-induced FLF at 4.5 h. All experiments were performed twice. Data is presented as mean ± s.e.m with n = 4 mice/group (Figure S1); *P<0.05 vs. PBS group (no Fas mAb treatment); *P<0.05 vs. NAC/Fas mAb-treated group. #*P*<0.05 vs PBS group (no Fas mAb treatment); #*P*<0.05 vs. PBS/Fas mAb-treated group. Analysis performed by one-way analysis of variance followed by Newman-Kuels post hoc test.
(TIF)
