# Supplementary figures and images for: The ROS Scavenger, NAC, Regulates Hepatic Vα14iNKT Cells Signaling during Fas mAb-Dependent Fulminant Liver Failure
Source: PLoS One. 2012 Jun 6;7(6):e38051. doi: 10.1371/journal.pone.0038051 (PMC3368940; doi:10.1371/journal.pone.0038051)

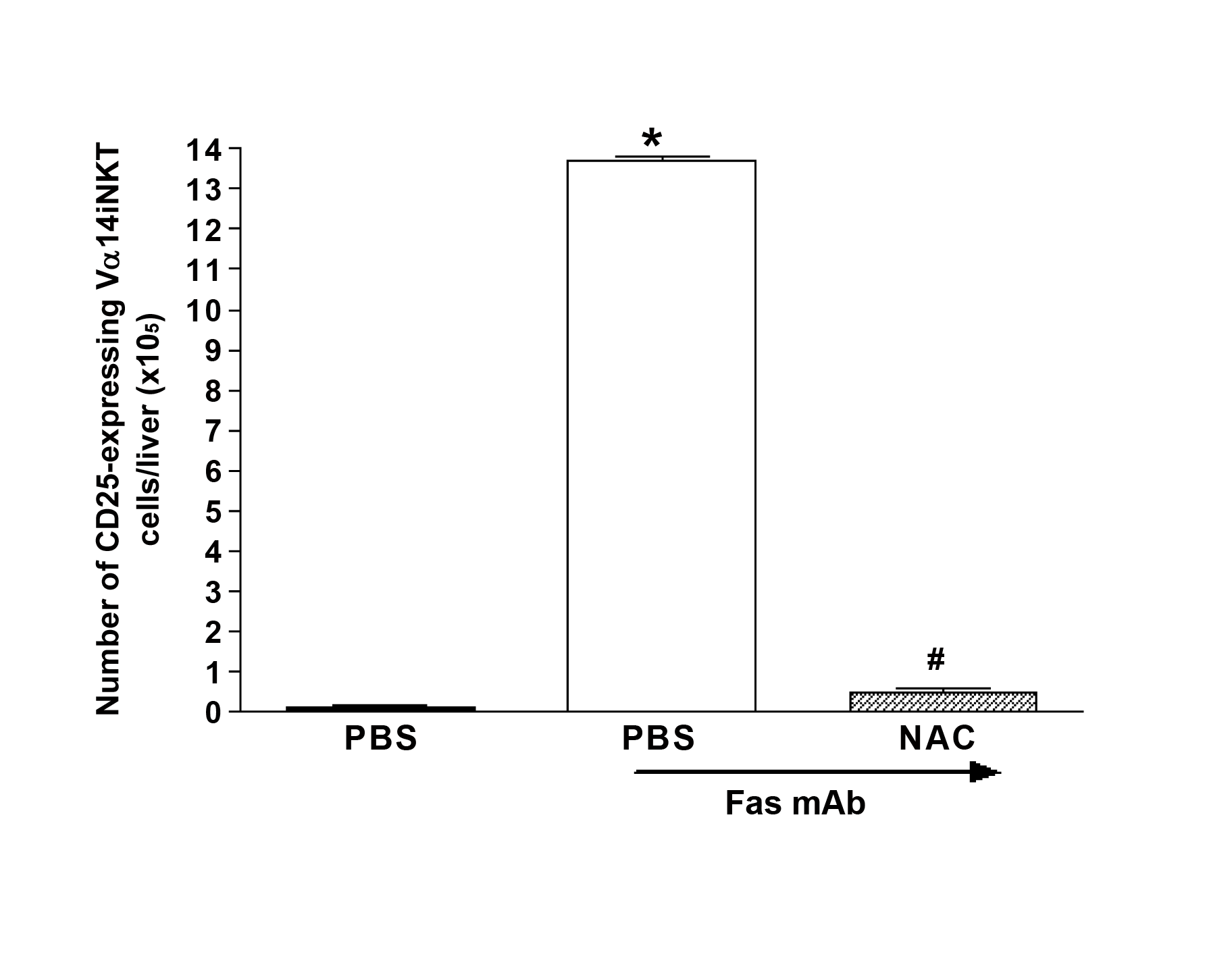

Supplement: Figure S1 — Effect of NAC treatment on intrahepatic Vα14 i NKT cell CD25 expression during agonistic Fas mAb-induced FLF. The number of CD25-expressing Vα14iNKT cells in the liver after PBS or NAC treatment during Fas mAb-induced FLF at 4.5 h. All experiments were performed twice. Data is presented as mean ± s.e.m with n = 4 mice/group (Figure S1); *P<0.05 vs. PBS group (no Fas mAb treatment); *P<0.05 vs. NAC/Fas mAb-treated group. #P<0.05 vs PBS group (no Fas mAb treatment); #P<0.05 vs. PBS/Fas mAb-treated group. Analysis performed by one-way analysis of variance followed by Newman-Kuels post hoc test. (TIF) [file pone.0038051.s001.tif]
